# Adenosine A_2A_ receptor availability in patients with early- and moderate-stage Parkinson’s disease

**DOI:** 10.1007/s00415-022-11342-1

**Published:** 2022-09-02

**Authors:** Imran Waggan, Eero Rissanen, Jouni Tuisku, Juho Joutsa, Semi Helin, Riitta Parkkola, Juha O. Rinne, Laura Airas

**Affiliations:** 1 Turku PET Centre, University of Turku, Itäinen Pitkäkatu 4A, 6th floor, 6007, 20520 Turku, Finland; 2 Division of Clinical Neurosciences, Turku University Hospital and University of Turku, Turku, Finland; 3 Department of Radiology, Turku University Hospital and University of Turku, Turku, Finland

**Keywords:** Parkinson’s disease, Adenosine A_2A_, Caudate nucleus, PET, Globus pallidus

## Abstract

**Introduction:**

Adenosine 2A (A_2A_) receptors co-localize with dopamine D_2 _receptors in striatopallidal medium spiny neurons of the indirect pathway. A_2A_ receptor activation in the striatum or pallidum decreases D_2 _signaling. In contrast, A_2A_ receptor antagonism may help potentiate it. Furthermore, previous PET studies have shown increased A_2A_ receptor availability in striatum of late-stage PD patients with dyskinesia. However, human in vivo evidence for striatal A_2A_ receptor availability in early-stage PD is limited. This study aimed to investigate possible differences in A_2A_ receptor availability in the striatum and pallidum of early- and moderate-stage PD patients without dyskinesias.

**Methods:**

Brain MRI and PET with [^11^C]TMSX radioligand, targeting A_2A_ receptors, was performed in 9 patients with early- and 9 with moderate-stage PD without dyskinesia and in 6 healthy controls. Distribution volume ratios (*DVR*) were calculated to assess specific [^11^C]TMSX binding in caudate, putamen and pallidum.

**Results:**

A_2A_ receptor availability (*DVR*) was decreased in the bilateral caudate of early-stage PD patients when compared with healthy controls (*P* = 0.02). Conversely, *DVR* was increased bilaterally in the pallidum of moderate-stage PD patients compared to healthy controls (*P* = 0.03). Increased mean striatal *DVR* correlated with higher motor symptom severity ($rho$ = 0.47, *P* = 0.02).

**Conclusion:**

Our results imply regional and disease stage-dependent changes in A_2A_ receptor signaling in PD pathophysiology and in response to dopaminergic medication.

**Supplementary Information:**

The online version contains supplementary material available at 10.1007/s00415-022-11342-1.

## Introduction

Parkinson’s disease (PD) is a multifaceted neurologic disorder characterized by degeneration of dopaminergic neurons in substantia nigra pars compacta (SNc). This decreases striatal dopamine, resulting in hyperactivity of the GABAergic medium spiny neurons (MSN) of the indirect pathway. It also causes an imbalance of striatal output from both the direct and indirect pathways. This functional imbalance causes motor symptoms in PD [1].

Adenosine 2A (A_2A_) receptors co-localize with dopamine D_2_ receptors at the somato-dendritic and nerve terminal level of striatopallidal MSNs of the indirect pathway [2, 3]. They have a homogenous distribution throughout the striatum and are also expressed in the external segment of globus pallidus [2, 4]. Activation of A_2A_ receptors at the dendritic level in the striatum or at the nerve terminal level in globus pallidus externa leads to a decrease in dopaminergic D_2_ signaling via antagonistic allosteric interaction between these two receptors [5, 6]. Clinical relevance of the interaction between A_2A_ and D_2_ receptors in PD is best established in advanced stage of the disease, where it forms the basis of the use of A_2A_ antagonists as an add-on medication with levodopa/carbidopa to reduce motor complications, especially the duration of ‘off’ periods in US and Japan [7]. PD patients also exhibit non-motor symptoms such as cognitive deficits and mood-related alterations already early in the disease [8, 9]. Pre-clinical studies based on animal PD models suggest an improvement in working memory impairments and depression like behavior following A_2A_ receptor blockade [10, 11]. A_2A_ antagonists have been shown to decrease cellular death resulting from alpha-synuclein toxicity [12]. It has also been postulated that the effect of A_2A_ antagonists on motor symptoms in early stages of PD may be stronger as compared to advanced stages because of the higher number of viable dopaminergic neurons [13].

Imaging the availability of A_2A_ receptors in vivo in humans is possible using positron emission tomography (PET). However, only a few studies of A_2A_ receptor PET imaging in PD have been reported to date [14–16]. These studies have provided preliminary evidence, indicating that the availability of A_2A_ receptors in caudate and putamen is increased in late-stage PD patients with dyskinesia compared to PD patients without dyskinesia and to healthy controls [14, 15]. Also, one of these studies showed an increase in putaminal A_2A_ receptor availability after the initiation of dopaminergic medication in drug-naïve PD patients [14]. Finally, despite the involvement of pallidum in PD pathophysiology, pallidal A_2A_ receptor availability has not been reported with PET at any stage of the disease [17]. We, therefore, sought to explore A_2A_ receptor availability in relevant brain regions of early- and moderate-stage PD patients and compared to healthy controls using PET imaging and [^11^C]TMSX radioligand which binds selectively to A_2A_ receptors [3].

## Methods

### Study protocols and patient consent

The protocol for this academic, investigator-initiated, cross-sectional study was approved by the Ethics Committee of the Hospital District of Southwest Finland. All study participants provided signed informed consent and the study was conducted in accordance with the World Medical Association’s declaration of Helsinki.

### Study participants

Ten patients with early stage, ten with moderate-stage idiopathic PD and six healthy controls were enrolled for this study. All patients fulfilled the clinically defined criteria for idiopathic Parkinson’s Disease without dyskinesia [18]. PD was considered to be at early stage if time from diagnosis was < 5 years, and at moderate stage if time from diagnosis was between 5 and 15 years. PD cohorts were recruited from the outpatient neurology clinics of Turku University Hospital and through study advertisements published on the Finnish Parkinson Foundation forums. Exclusion criteria included the presence of drug-induced dyskinesia, history of other neurological or psychiatric diseases, or another significant comorbidity. Demographics and clinical features of PD patients and healthy controls are summarized in Table 1. To avoid the possible short-term effects of dopaminergic medications on A_2A_ receptor availability, PET scans for PD patients were performed after 12 h (for standard levodopa) and 24 h (for prolonged release levodopa/carbidopa, MAO-B inhibitors and/or dopamine agonists) cessation of dopaminergic medication (Supplementary Table 1). Moreover, all study subjects were instructed to avoid consuming caffeinated beverages within 24 h before the scanning considering the antagonistic action of caffeine on A_2A_ receptors and half-life of caffeine of 2.3–9.9 h [19–21].

**Table 1:** Demographics and clinical parameters of healthy controls and PD patients

	Healthy controls (*n* = 6)	PD early stage (*n* = 9)	PD moderate stage (*n* = 9)	*P*-value
Sex (F/M)	5/1	4/5	5/4	0.318
Age (years)	60.6 (± 8.8)	65.1 (± 8.2)	67.1 (± 7.5)	0.336
Disease duration (years), median (range)	NA	1.8 (1.5–2.2)	8.4 (5.6–10.8)	** < 0.001**
Handedness (right/left) (*n*)	Not available	9/0	9/1	1.000
UPDRS I	NA	1.1 (± 1.7)	1.8 (± 1.3)	0.362
UPDRS II	NA	5.4 (± 2.3)	9.3 (± 3.9)	**0.020**
UPDRS III	NA	17.9 (± 5.9)	31.8 (± 2.7)	** < 0.001**
UPDRS IV	NA	1.7 (± 2.1)	1.4 (± 1.2)	0.785
UPDRS V	NA	2.0 (± 0.4)	2.5 (± 0.4)	**0.001**
LED (mg)	NA	196.8 (± 202.8)	727.3 (± 353.7)	** < 0.001**
LEDD_DA_ (mg)	NA	210.5 (± 234.3)	177.9 (± 59.0)	0.708
MMSE	Not available	28.9 (± 0.6)	28.6 (± 1.0)	0.408
Injection dose (MBq)	490.7 (± 25.1)	483.2 (± 26.6)	492.4 (± 21.0)	0.705
Radiochemical purity (%)	97.8 (± 0.4)	97.7 (± 0.5)	97.3 (± 0.5)	0.054
Injected mass (ug)	0.64 (± 0.3)	0.60 (± 0.3)	0.38 (± 0.2)	0.188
Molar activity (MBq/nmol)	350 (± 120)	410 (± 200)	570 (± 180)	0.277

Sex and handedness were compared using Fisher’s exact test. Age, injection dose, radiochemical purity and injected mass were compared using one-way ANOVA. Mann–Whitney *U* test was used for the rest of the parameters

*UPDRS* Unified Parkinson’s disease rating Scale, *LED* Levodopa equivalent dose, *LEDD*_*DA*_ Levodopa equivalent daily dose of dopamine agonists, *MMSE* mini mental status examination, *PD* Parkinson’s disease, *NA* not applicable

Bold values represent statistical significance

### Clinical assessment

All patients underwent a thorough medical and neurological examination prior to the PET scan. Definition of the disease duration, Unified Parkinson’s disease rating scale (UPDRS) I–V scoring, Mini Mental Status Examination (MMSE) and calculation of the Levodopa Equivalent Dose (LED) were acquired as part of the examination.

### [^11^C]TMSX radioligand production and administration

Production of [^11^C]TMSX radioligand was performed according to the methodology described in detail in our earlier study [22]. The radiochemical purity of the produced radioligand was 97.6 (± 0.5) percent. At the start of the dynamic PET scan, a smooth single bolus of [^11^C]TMSX was injected into the left antecubital vein and was subsequently flushed with saline. The mean (± SD) injected dose (MBq) of [^11^C]TMSX for controls, and for early-stage and moderate-stage PD patients was 490 (± 25.1), 483 (± 26.6) and 492 (± 21.0), respectively, without significant differences in doses between the groups (*P* = 0.705). One patient from the early PD group and one from moderate PD group were excluded from the final analyses, since the [^11^C]TMSX PET data were not reliably quantifiable due to the relatively low injected dose of the radiotracer and high injected mass, respectively.

### PET and MR acquisition and image processing

A 60-min dynamic brain PET scan with [^11^C]TMSX radiotracer was acquired using the ECAT HRRT scanner (Knoxville, USA). Tissue attenuation maps were obtained with a 6-min transmission scan for attenuation correction using ^137^Cs point source prior to the dynamic scan. Twenty-seven timed frames (6 × 10, 1 × 30, 5 × 60, 5 × 150, and 8 × 300 s) lasting a total of 3600 s were used for PET image reconstruction in accordance with methods described in our earlier study [22].

All subjects underwent brain MRI with 1.5T Nova Dual scanner (Philips, Netherlands). Axial 3D T1 weighted images were used as anatomic reference for PET images. The T1 images were co-registered with motion corrected dynamic PET scans, where the image processing was carried out using Statistical Parametric Mapping software (SPM12, The Wellcome Centre for Human Neuroimaging, UCL, London) as detailed in our earlier study [23]. For the evaluation of region-specific radioligand binding, reference tissue input Logan graphical analysis within 20–60-min interval was used to estimate distribution volume ratios (*DVR*). This was applied to region of interest (ROI)-based time-activity curves (TAC) using clustered cerebral grey matter as a reference region acquired with a supervised clustering algorithm (modified from the Super PK software) [24]. The supervised clustering algorithm assumes that each dynamic PET data TAC is a combination of four kinetic class TACs that correspond to healthy grey matter, white matter, blood and high specific binding gray matter. In this study, kinetic class TACs corresponding to normal [^11^C]TMSX binding in grey matter, white matter and blood were first defined from a healthy volunteer group (*n* = 7). The kinetic class TAC corresponding to high specific [^11^C]TMSX binding was acquired from thalamus in multiple sclerosis patients (*n* = 12) and was compared with anterior putamen binding in PD patients (*n* = 9), as described in [23]. Next, the contribution of each kinetic class to the voxel-level [^11^C]TMSX TACs was estimated by using non-negative least squares estimation. Lastly, the clustered reference region TAC was calculated from the dynamic [^11^C]TMSX PET data as a weighted average by using the grey matter coefficients as weighting factors. Parametric maps for voxel-level analysis were generated using non-displaceable binding potential (*BP*_*ND*_) images using basis function implementation of simplified reference tissue model (SRTM) with 250 basis functions, and by using the values 0.06 and 0.8 for the lower and upper limit for θ_3_.

### Region of interest and voxel-level analyses of the [^11^C]TMSX PET data

For the ROI analyses, Freesurfer software (v6.0, http://surfer.nmr.mgh.harvard.edu/) was used for anatomical parcellation of the 3D T1 MR images to extract region-specific *DVR* values from the co-registered PET scans [25]. Analyses were limited to three bilateral ROIs, i.e., caudate, putamen and pallidum for the primary outcome.

Mean *DVR* values of the whole dorsal striatum were calculated using a combined mask of caudate and putamen, to check associations with clinical parameters following previous studies [15]. To assess the within and between-group effect of lateralization, contralateral and ipsilateral ROIs were drawn manually for caudate and putamen using in-house software (Carimas v2.9). These unilateral ROIs were defined as contralateral or ipsilateral with respect to each patient’s clinically more affected side, based on the respective unilateral sums of the UPDRS III scores. Comparison of *DVR*s in ipsilateral or contralateral ROIs of the PD groups with healthy controls was carried out against the combined average *DVR* of the left and right striatal ROIs of controls.

Parametric [^11^C]TMSX *BP*_*ND*_ images were used to evaluate voxel-wise differences between healthy controls and patients with early- and moderate-stage PD utilizing SPM12. Images were smoothed with 3D Gaussian 4 mm FWHM filter to improve signal-to-noise ratio and ensure normal data-distribution before voxel-wise analyses.

### Statistical methods

ROI-level statistical analyses were performed with GraphPad Prism software (v9.1, California, USA). Normality of distribution was checked graphically and using the Shapiro–Wilk test. Differences in categorical variables were evaluated using non-parametric Fischer’s exact test. Mann–Whitney *U* test was used to assess differences in disease duration between patients with early- vs. moderate-stage PD. One-way ANOVA was utilized to evaluate differences in age, molar activity, injected mass, radiochemical purity and injected radioligand dose between groups. To investigate local changes at the ROI level, an independent *t*-test was used with Bonferroni correction for multiple comparisons applied afterward. Paired t-test was utilized to calculate the within-group differences of ipsilateral and contralateral sides determined by the clinically more affected side of the patient. Cohen’s d was calculated to measure the effect size for statistically significant comparisons. Spearman test was used to evaluate correlations between clinical parameters and striatal and pallidal [^11^C]TMSX *DVR* values. *P-*values < 0.05 from two-tailed tests were considered statistically significant in ROI-level analyses.

For the voxel-level analyses in SPM12, a two-sample *t*-test was carried out using parametric *BP*_*ND*_ image with overlaid striatal mask, including pallidum, in MNI-152 space. The cluster-based inference with cluster-defining threshold of *P* = 0.001 (*T* = 3.85) was used, and the *P* < 0.05 family-wise error-corrected critical cluster size was 115 voxels.

## Results

No differences were observed in the age (*P* = 0.336) and sex distribution (*P* = 0.318) between healthy control and early- and moderate-stage PD groups. Patients with moderate-stage PD had a longer disease duration (*P* < 0.001), higher UPDRS III score (*P* < 0.001) and LED (*P* < 0.001) compared to the early-stage PD patients (Table 1).

### ROI-based analyses

**Fig. 1:**
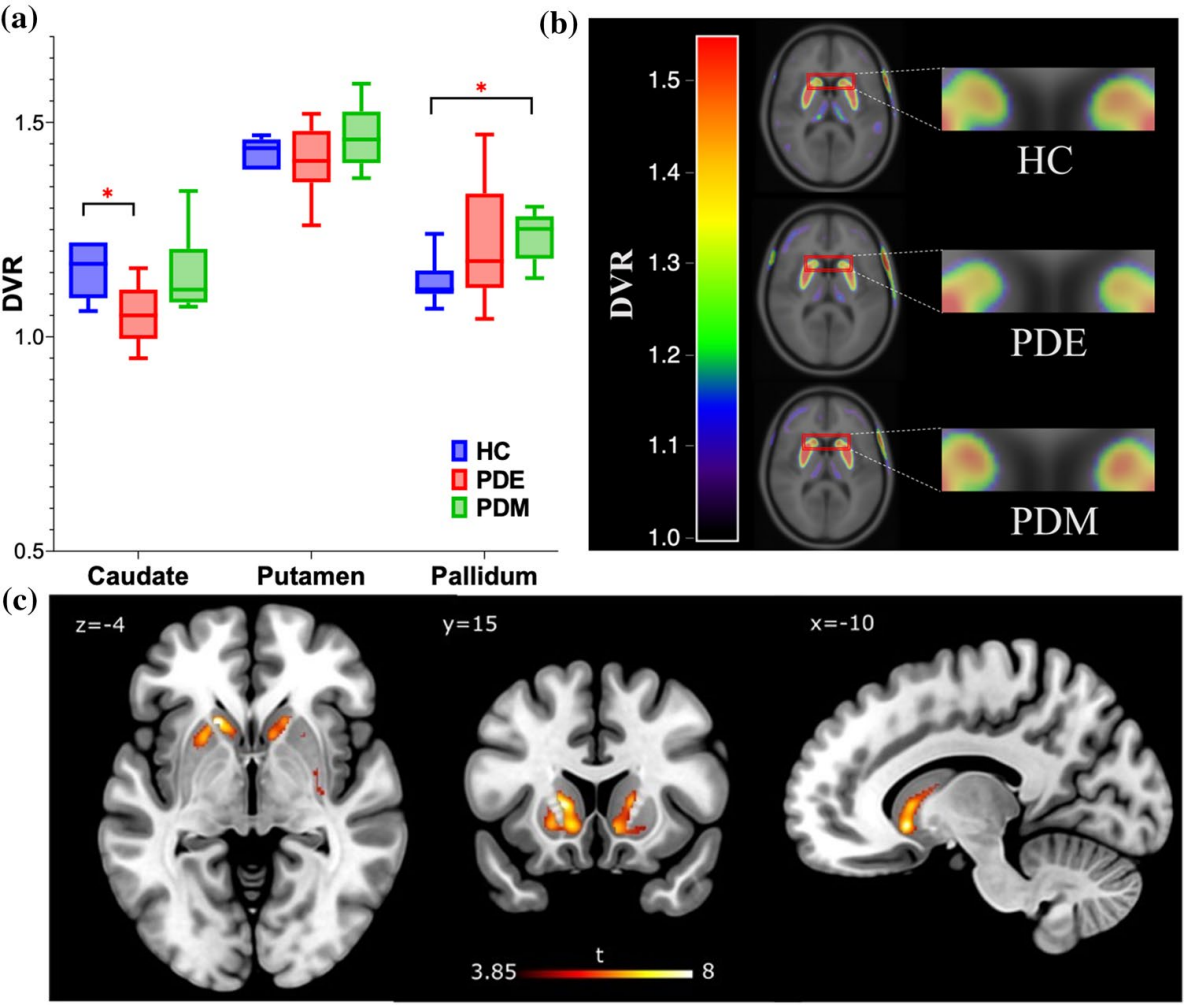
**a** Boxplot with distribution volume ratio (*DVR*) values representing [^11^C]TMSX binding in adenosine A_2A_ receptors in the striatum of healthy control (HC), Parkinson's disease early-stage (PDE) and Parkinson's disease moderate-stage (PDM) groups. The availability of A_2A_ receptors is decreased in bilateral caudate nucleus in PDE (*P* = 0.02, Independent *t*-test) when compared to HC. A_2A_ receptor availability in bilateral pallidum in PDM is increased when compared to HC (*P* = 0.03, Independent *t*-test). *P < 0.05 was considered statistically significant after correction for multiple comparison. **b** Mean parametric *DVR* image of HC, PDE and PDM patients overlaid on MNI 152 template demonstrating A_2A_ receptor availability in the striatum with the binding in the head of caudate nucleus enlarged. **c** Voxel-level analyses depicting a decrease in *BP*_*ND*_ in clusters defining bilateral caudate (*P* < 0.001) of early PDE patients when compared with HC. Decreases can also be observed in left (and to a lesser extent right) anterior putamen of PDE patients vs HCs. The cluster-based inference with cluster-defining threshold of *P* = 0.001 (*T* = 3.85) was used and the *P* < 0.05 family-wise error-corrected critical cluster size was 115

**Fig. 2:**
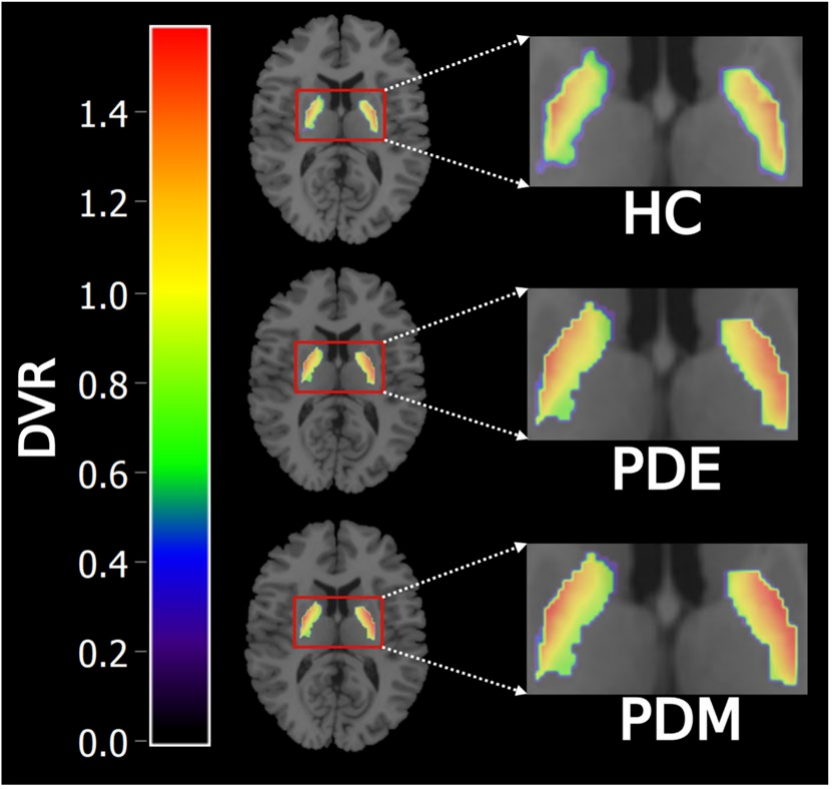
Mean parametric *DVR* pallidum mask of healthy controls (HC), Parkinson's disease early-stage (PDE) and Parkinson's disease moderate-stage (PDM) patients overlaid on ch2better template demonstrating higher A_2A_ receptor availability in PDM as compared to HC (*P* = 0.03, Independent *t*-test). *P* < 0.05 was considered statistically significant after correction for multiple comparisons

ROI-level analyses revealed that patients with early-stage PD had lower mean [^11^C]TMSX *DVR* in bilateral caudate when compared to healthy controls (effect size [ES] = 1.64, *P* = 0.02) (Fig. 1a, b). No differences were observed in the bilateral caudate *DVR*s of early-stage patients when compared to moderate-stage patients (*P* = 0.14), and in the bilateral caudate *DVR*s of the patients with moderate stage when compared to healthy controls (*P* > 0.99). Conversely, an increase in mean [^11^C]TMSX *DVR* in bilateral pallidum was observed in moderate-stage PD when compared with healthy controls (ES = 1.64, *P* = 0.03), whereas no difference was observed in the bilateral pallidum when comparing early-stage PD group with moderate-stage PD (*P* > 0.99) and healthy controls (*P* = 0.64) (Fig. 2). No significant differences were observed in bilateral putamen *DVR*s in the group comparisons of early-stage PD vs. controls *P* > 0.99, moderate-stage PD vs. controls (*P* = 0.74) and early-stage PD vs. moderate-stage PD (*P* = 0.42) (Fig. 1a).

In the evaluation of the unilateral striatal ROIs in relation to the clinically more affected (contralateral) and less affected (ipsilateral) sides among the PD patients, significant decrease was observed in the ipsilateral caudate of early-stage PD patients (ES = 1.76, *P* = 0.01) compared to the average caudate *DVR* of healthy controls. Similar, but non-significant trend was observed for the comparison of contralateral caudate of early-stage PD to controls, (ES = 1.38, *P* = 0.06). No difference was observed in the ipsilateral vs. contralateral within-group comparison of caudate in the early-stage PD patients (Table 2b). Similarly, an increase in the uptake of [^11^C]TMSX in ipsilateral pallidum of moderate-stage PD patients when compared to healthy controls was noted (ES = 1.83, *P* = 0.02) but not for the contralateral pallidum (*P* = 0.92). Again, no difference was observed in the ipsilateral vs. contralateral region of interest comparison within the PD moderate group (Table 2b).

**Table 2:** (a) Average (mean ± SD) distribution volume ratio (DVR) values representing [^11^C]TMSX binding to adenosine A_2A_ receptors in whole, ipsilateral (ipsi) and contralateral (contra; relative to the clinically more affected side) striatal structures of healthy control (HC), Parkinson’s disease early stage (PDE) and Parkinson’s disease moderate-stage (PDM) groups, (b) Within and between-group comparison of ipsilateral and contralateral striatal structures reported with (without) Bonferroni correction

	Caudate	Putamen	Pallidum
(a)			
HC (avg left + right), mean ± SD	1.163 ± 0.063	1.435 ± 0.030	1.135 ± 0.059
PDE (avg left + right), mean ± SD	1.056 ± 0.056	1.411 ± 0.080	1.217 ± 0.139
PDE ipsi, mean ± SD	1.054 ± 0.061	1.421 ± 0.067	1.234 ± 0.169
PDE contra, mean ± SD	1.080 ± 0.061	1.400 ± 0.125	1.193 ± 0.122
PDM (avg left + right), mean ± SD	1.146 ± 0.093	1.472 ± 0.071	1.231 ± 0.058
PDM ipsi, mean ± SD	1.151 ± 0.100	1.454 ± 0.075	1.239 ± 0.059
PDM contra, mean ± SD	1.147 ± 0.094	1.496 ± 0.091	1.223 ± 0.086
(b)			
HC left vs right, (*P*)	> 0.99 (0.77)	> 0.99 (0.62)	> 0.99 (0.59)
PDE ipsi vs HC, (*P*)	**0.01 (0.005)**	> 0.99 (0.64)	0.57 (0.18)
PDE contra vs HC, (*P*)	0.06 **(0.02)**	> 0.99 ((0.52)	> 0.87 (0.29)
PDE ipsi vs contra, (P)	> 0.99 (0.39)	> 0.99 (0.66)	> 0.99 (0.56)
PDM ipsi vs HC, (*P*)	> 0.99 (0.50)	> 0.99 (0.59)	**0.02 (0.006)**
PDM contra vs HC, (P)	> 0.99 (0.52)	0.92 (0.31)	0.92 (0.06)
PDM ipsi vs contra, (*P*)	> 0.99 (0.92)	0.93 (0.30)	> 0.99 (0.66)

*P* < 0.05 was considered statistically significant

Independent *t*-test was used for statistical comparison

**Fig. 3:**
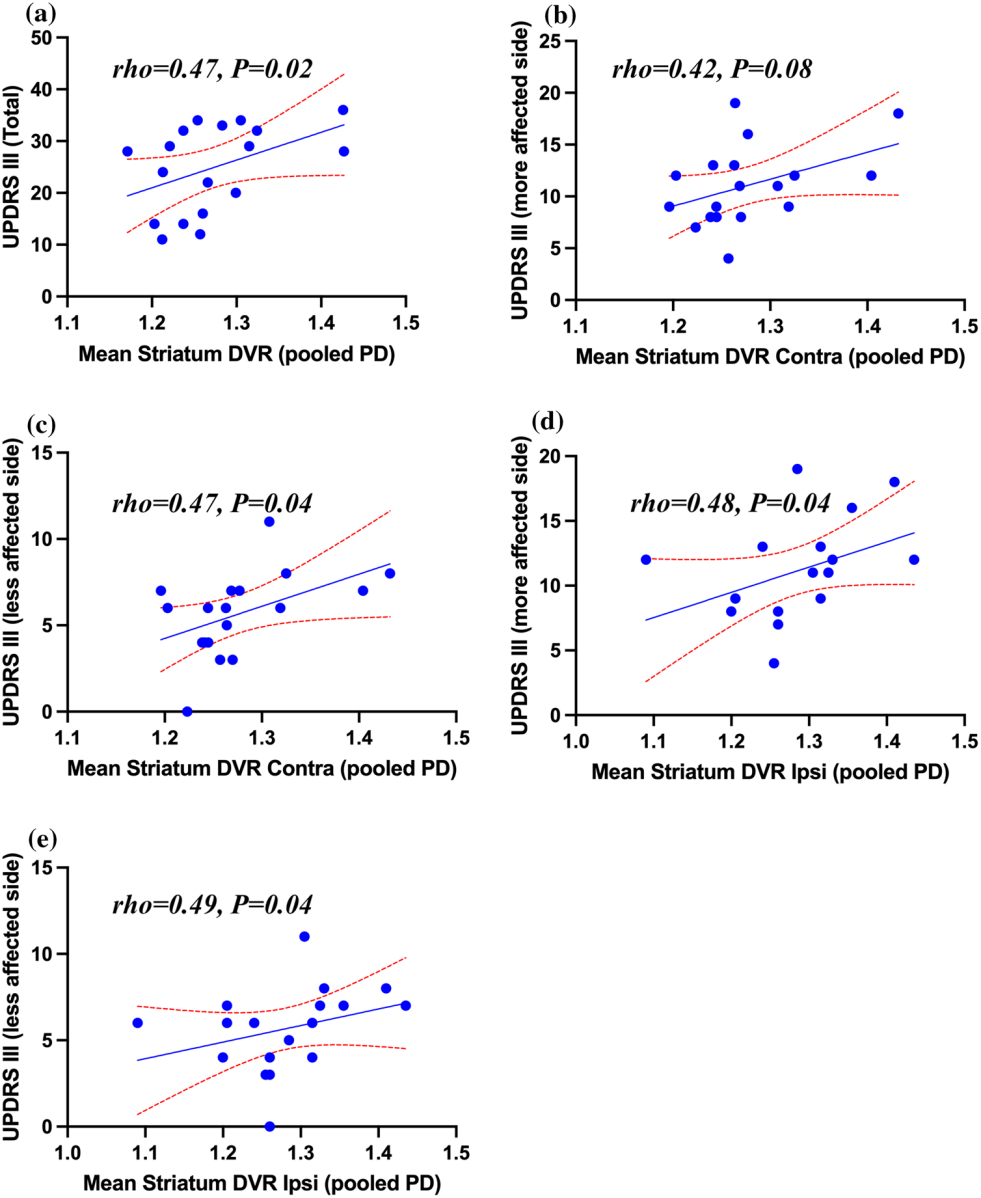
Scatter plots showing associations between [^11^C]TMSX distribution volume ratio (*DVR*) representing adenosine A_2A_ receptor availability in whole, ipsilateral (Ipsi) and contralateral (Contra; relative to the clinically more affected side) striatum and UPDRS III scores of pooled Parkinson’s disease (PD) patients (PD early + PD moderate). **a** Correlation between mean bilateral striatum *DVR* of pooled PD patients and their total UPDRS III score. **b** Correlation between mean contralateral striatum *DVR* of pooled PD patients and the UPDRS III score of their clinically more affected side. **c** Correlation between mean contralateral striatum *DVR* of pooled PD patients and the UPDRS III score of their clinically less affected side. **d** Correlation between mean ipsilateral striatum *DVR* of pooled PD patients and the UPDRS III score of their clinically more affected side. **e** Correlation between mean ipsilateral striatum *DVR* of pooled PD patients and the UPDRS III score of their clinically less affected side. UPDRS: Unified Parkinson’s disease rating scale

The mean striatal [^11^C]TMSX *DVR* correlated with UPDRS III scores ($rho$ = 0.47, *P* = 0.02) within the pooled group of PD patients (Fig. 3a). However, no correlations were observed between the mean *DVR*s separately in caudate, putamen, pallidum and the UPDRS III scores (Table 3). On the other hand, we noticed a moderate association between [^11^C]TMSX binding in striatum contralateral to the side with predominant motor symptoms and the UPDRS III score of the clinically less affected side ($rho$ = 0.47, *P* = 0.04) and a similar but non-significant trend with the UPDRS III score of the clinically more affected side ($rho$ = 0.42, *P* = 0.08) in the pooled PD group (Fig. 3b, 3c). Moderate correlation was also observed between [^11^C]TMSX *DVR* in the striatum ipsilateral to the side with predominant motor symptoms and the UPDRS III score of the clinically more affected side of the body ($rho$ = 0.48, *P* = 0.04) as well as with the UPDRS III score of the clinically less affected side ($rho$ = 0.49, *P* = 0.04) in pooled group of PD patients (Fig. 3d, e).

**Table 3:** Spearman correlations between distribution volume ratio (DVR) values representing [^11^C]TMSX binding to adenosine A_2A_ receptors of whole striatum, caudate, putamen and pallidum of all patients with Parkinson’s disease (*pooled*), Parkinson’s disease early stage (PDE) and Parkinson’s disease moderate-stage (PDM) patients and their disease duration, UPDRS III and LED

	Disease duration *rho* (*P*)	UPDRS III *rho* (*P*)	LED *r*ho (*P*)
**Striatum** (pooled)	0.40 (0.05)	**0.47 (0.02)**	0.38 (0.06)
Striatum PDE	– 0.43 (0.13)	0.01 (0.49)	**– 0.68 (0.02)**
Striatum PDM	0.29 (0.24)	– 0.03 (0.47)	0.28 (0.23)
**Caudate** (pooled)	0.46 (0.07)	0.46 (0.06)	**0.55 (0.02)**
Caudate PDE	0.25 (0.51)	0.22 (0.57)	0.12 (0.77)
Caudate PDM	0.17 (0.69)	0.24 (0.54)	0.47 (0.21)
**Putamen** (pooled)	0.17 (0.52)	0.22 (0.78)	0.07 (0.78)
Putamen PDE	**– 0.82 (0.01)**	– 0.24 (0.53)	**– 0.76 (0.02)**
Putamen PDM	0.35 (0.40)	– 0.22 (0.57)	0.17 (0.68)
**Pallidum** (pooled)	– 0.13 (0.62)	0.06 (0.79)	– 0.10 (0.70)
Pallium PDE	– 0.66 (0.06)	– 0.35 (0.35)	– 0.42 (0.26)
Pallidum PDM	– 0.08 (0.85)	0.36 (0.34)	0.00 (> 0.99)

*UPDRS* Unified Parkinson’s disease rating scale, *LED* Levodopa equivalent dose

Bold values represent statistical significance

**Fig. 4:**
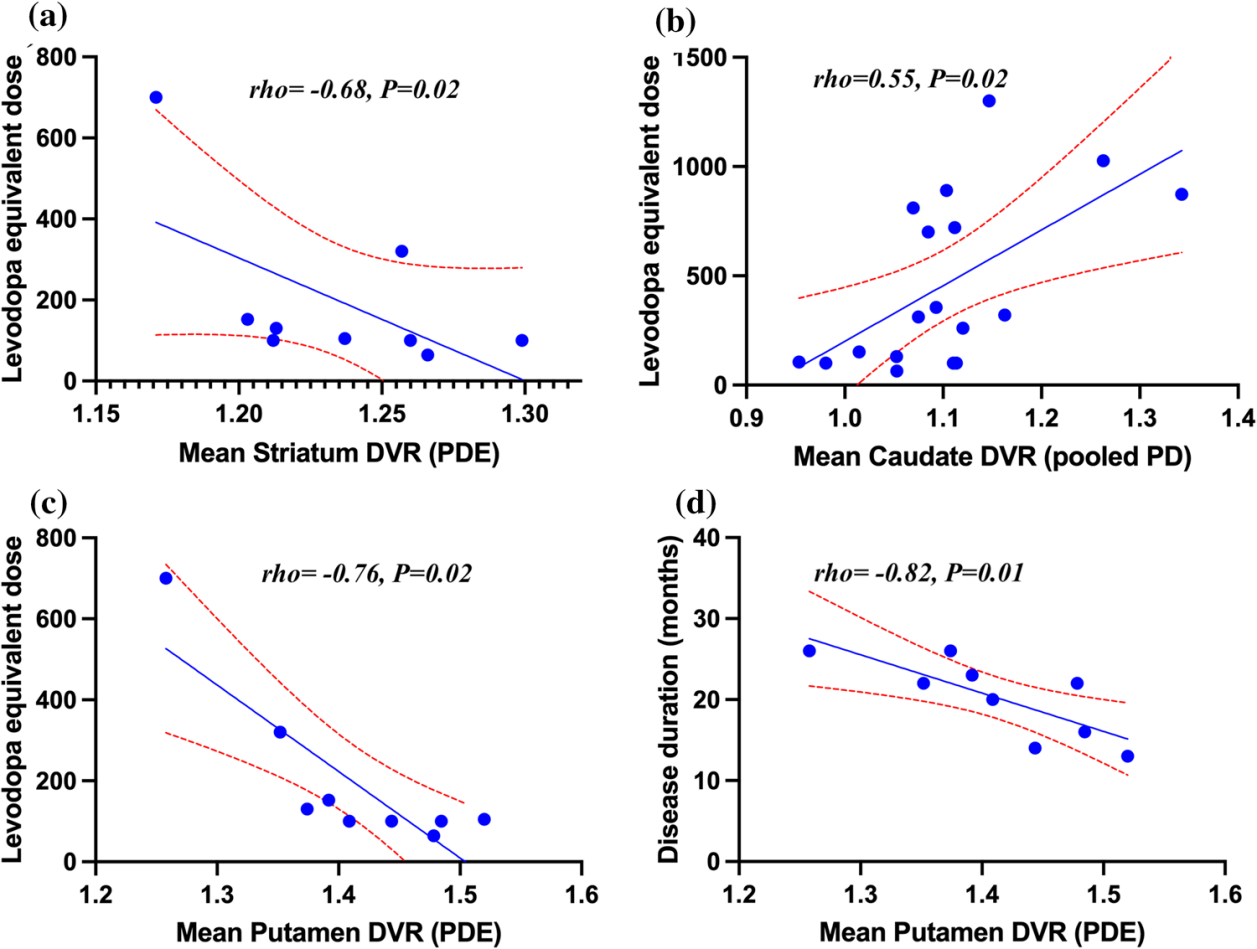
Scatter plots showing associations between [^11^C]TMSX distribution volume ratio (*DVR*) representing adenosine A_2A_ receptor availability in striatal regions of interest and clinical parameters of Parkinson’s disease (PD) patients. **a** Correlation between mean bilateral striatum *DVR* of PD early-stage (PDE) patients and their Levodopa equivalent dose (LED). **b** Correlation between mean bilateral caudate *DVR* of pooled PD patients (PD early-stage + PD moderate-stage patients) and their LED. **c** Correlation between mean bilateral putamen *DVR* of PDE patients and their LED. **d** Correlation between mean bilateral putamen *DVR* of PDE patients and their disease duration

We also observed an association between striatal [^11^C]TMSX binding and levodopa equivalent dose (LED) in patients with early-stage PD ($rho$ = – 0.68, *P* = 0.02) (Fig. 4a). Mean bilateral caudate [^11^C]TMSX *DVR* also correlated with LED in pooled PD patients ($rho$ = 0.55, *P* = 0.02) (Fig. 4b). Similarly, a strong correlation was found between [^11^C]TMSX binding in bilateral putamen and LED ($rho$ = – 0.76, *P* = 0.02) as well as with disease duration ($rho$ = – 0.82, *P* = 0.01) in early-stage PD patients (Table 3, Fig. 4c and 4d).

### Voxel-level analyses

Voxel-level analyses showed decreased *BP*_*ND*_ in clusters defining bilateral caudate (*P* < 0.001) of early-stage PD patients when compared with healthy controls (Fig. 1c). Similarly, decreased *BP*_*ND*_ was also observed in left anterior putamen, and to a lesser extent in right anterior putamen of early-stage PD subjects when compared to controls. However, no differences were observed when comparing moderate-stage PD patients with healthy controls and early-stage PD patients (data not shown).

## Discussion

Our work demonstrates that A_2A_ receptor availability is significantly decreased in the caudate in early-stage PD patients when compared to healthy controls. Furthermore, an increased availability of adenosine A_2A_ receptors in the pallidum in moderate-stage PD patients was observed compared to healthy controls.

A decrease in A_2A_ receptor availability in the bilateral caudate of early-stage PD patients was evident both in the region of interest as well as the voxel-level analyses (Fig. 1). This is in line with evidence from pre-clinical and post-mortem PD studies, suggesting a decrease of A_2A_ receptors in the caudate nucleus but not in the putamen [26–28]. Interestingly, [^11^C]Raclopride studies targeting dopamine D_2_ receptors, which co-localize with A_2A_ receptors in striatum, have also shown a decrease in binding in caudate of early-stage PD patients while binding in the putamen was similar to healthy volunteers [29, 30]. Experimental studies demonstrate negative allosteric modulation of A_2A_ receptor binding by D_2_ agonists such as pramipexole and rotigotine [31]. Previously, we have shown that unlike in putamen and pallidum, the repeatability of [^11^C]TMSX binding in caudate and its subregions is poor when comparing *DVR* values during and after a short-term (12–24 h) cessation of routine dopaminergic medication [32]. Our current results also suggest a correlation between higher LED and higher [^11^C]TMSX binding in the caudate of pooled PD patients. On the other hand, we have shown an association between higher LED and lower [^11^C]TMSX binding in striatum and putamen of early-stage patients. Taking all these findings into consideration, we propose that dopaminergic medication affects A_2A_-D_2_ heterodimer in caudate differentially as compared to the rest of striatum. This selective reduction could be attributed to the regional differentiation in the subpopulation of A_2A_ receptors or to the relatively heavier reliance on dopamine agonists in earlier stages of the disease (Supp Table 1) [30]. Previous studies suggest that the degeneration of dopaminergic neurons projecting to putamen precedes those projecting to caudate [33]. This difference in dopamine may also explain the decrease in A_2A_ receptor availability in caudate and not putamen of early PD patients. Another possible, but less likely, explanation could be the degeneration of dendritic spines of striatopallidal MSNs, where A_2A_ receptors are heavily expressed, as the disease progresses [34].

We also observed an increase in bilateral pallidal availability of A_2A_ receptors in the moderate stage of the disease at ROI level compared with healthy controls (Fig. 2). However, the result could not be replicated at the voxel-level likely due to the high level of noise in dynamic PET scan TACs and the strict cluster-defining threshold. Within the pallidum, A_2A_ receptors are mainly located on the nerve terminals of GABAergic striatopallidal neurons in the external segment of globus pallidus [2, 4, 35]. A_2A_ receptor-mediated modulation of the indirect pathway output occurs not only at the striatal level but also at the pallidal nerve terminals [36]. However, this ‘dual excitatory modulation’ is relatively less potent in globus pallidus externa as compared to striatal A_2A_ receptors [37]. Our result is in agreement with previous post-mortem evidence where binding of A_2A_ receptor specific ligand was found to be higher in the external segment of globus pallidus of postmortem brains of PD patients compared to controls [27]. Recent evidence suggests an increase in ATP in nigrostriatal pathway and CD73-mediated increase in adenosine within striatum [38, 39]. This extracellular increase in adenosine could explain the compensatory increases in pallidal A_2A_ receptor availability in moderate PD patients in our study and also the increase in striatal A_2A_ receptor binding in PD patients with dyskinesia reported earlier [14, 15]. Increased receptor expression invariably results in higher G protein-coupled receptor (GPCR) activity [40]. This implies that higher pallidal A_2A_ receptor expression could possibly counteract the effect of antiparkinsonian therapeutics on indirect pathway in moderate-stage patients. It is imperative to note that therapeutic modulation of the indirect pathway is more important to reduce motor disability in PD as compared to the direct pathway [1]. Therefore, higher pallidal expression of A_2A_ receptors can be one potential mechanism contributing to the decreased dose-related efficacy of dopaminergic medication in PD patients during disease progression [41]. Increased A_2A_ receptor expression may also alter output of globus pallidus externa to other basal ganglia structures as it innervates the striatum, subthalamic nucleus, globus pallidus interna and substantia nigra pars reticulata [42].

Our data also demonstrate that the decrease in [^11^C]TMSX binding in caudate of early-stage PD patients and the increase in pallidum of moderate-stage patients is slightly more pronounced on the ipsilateral side with respect to predominant motor symptoms. A significant association was shown between [^11^C]TMSX binding of bilateral striatum and UPDRS III scores of pooled PD patients. Similarly, we also observed moderate association of ipsilateral striatal [^11^C]TMSX binding with the UPDRS III score of the more and less affected side. Contrarily, the binding of contralateral striatum had a moderate association with the UPDRS III score of the clinically less affected side only. An earlier [^11^C]TMSX PET study found an asymmetric decrease in putaminal binding in the more affected side when compared with the less affected side in drug naïve patients [14]. Similarly, an increasing number of PET studies examining dopaminergic degeneration have shown a more pronounced deficit in the contralateral striatum. However, some studies report it to be higher in the ipsilateral striatum relative to the clinically more affected side of the body possibly due to ‘floor effect’[43–45]. This factor may also be responsible for the higher change in binding on the ipsilateral side in our study [46].

We also found a strong correlation between lower bilateral putaminal [^11^C]TMSX binding and higher disease duration in early-stage patients. However, no correlation was found between disease duration and striatal or pallidal binding in moderate-stage patients. This inconsistency could potentially be attributed to compensatory increase in A_2A_ receptor expression in later stages of the disease [14, 15]. Furthermore, since our findings are based on a limited number of patients and we lack the imaging data of the dopaminergic system, the results from our study should be considered preliminary [43]. In addition, the low signal-to-noise ratio of [^11^C]TMSX should also be considered a potential limitation of our work. Instead of using centrum semiovale as reference region or the ‘gold standard’ arterial blood sampling, we used clustered cerebral grey matter as reference region for our work [47]. This method was validated and found to be as robust as arterial blood sampling for use with [^11^C]TMSX in our earlier work [23, 32].

*Mishina and colleagues*[14] found an asymmetric decrease in [^11^C]TMSX binding in the putamen of clinically more affected side as compared to less affected side in drug naïve patients. The availability of A_2A_ receptors in bilateral putamen increased in the same patients approximately 15 months after starting regular dopaminergic medication [14]. However*,* they did not report of a comparison to healthy controls in these patients after the period of dopaminergic medication usage, so the inference on the magnitude of longitudinal change remains limited.

In conclusion, we have demonstrated that the availability of A_2A_ receptors is decreased in the caudate in patients with early-stage PD and increased in the pallidum in patients with moderate-stage PD. Further studies are warranted to confirm these findings and to investigate A_2A_-related pathological changes in PD.

## Supplementary Information

Below is the link to the electronic supplementary material.

Supplementary file1 (DOCX 13 KB)

## Data Availability

The raw data used in the preparation of this article can be shared in anonymized format by request of a qualified investigator.
