# Supplementary material for: Adenosine A2A receptor availability in patients with early- and moderate-stage Parkinson’s disease
Source: J Neurol. 2022 Sep 2;270(1):300–10. doi: 10.1007/s00415-022-11342-1 (PMC9813038; doi:10.1007/s00415-022-11342-1)
Supplement: Supplementary file 1 — Supplementary file1 (DOCX 13 KB) [file 415_2022_11342_MOESM1_ESM.docx]

**Supplementary Table 1.** Antiparkinsonian therapy prescribed to Parkinson’s disease (PD) early and moderate stage patients

|  | ***PD Early***  ***Stage (n)*** | ***PD Moderate***  ***Stage (n)*** |
| --- | --- | --- |
| Dopamine agonists only | 2 | 0 |
| MAO-B inhibitors only | 2 | 0 |
| Dopamine agonists + MAO-B inhibitors | 3 | 1 |
| Dopamine agonists + Levodopa/Carbidopa | 0 | 1 |
| MAO-B inhibitors + Levodopa/Carbidopa | 1 | 0 |
| Dopamine agonists + MAO-B inhibitors + Levodopa/Carbidopa | 1 | 7 |
